# Disease Progression and Death From Cervical Melanoma in a Patient Undergoing Nivolumab Therapy: A Case Report

**DOI:** 10.7759/cureus.52811

**Published:** 2024-01-23

**Authors:** Mateus Lopes Macêdo, Rafael Everton Assunção Ribeiro da Costa, Cristiane Amaral dos Reis, Raimundo Gerônimo Da Silva Júnior, Ary Oliveira Pires, Sabas Carlos Vieira

**Affiliations:** 1 Medicine, Uninovafapi University Center, Teresina, BRA; 2 Health Science Center, State University of Piauí, Teresina, BRA; 3 Clinical Oncology, Oncocenter, Teresina, BRA; 4 Health Science Center, Federal University of Piauí, Teresina, BRA; 5 Radiology, UDI 24-Hour Clinic, Teresina, BRA; 6 Tocogynecology, Oncocenter, Teresina, BRA

**Keywords:** case reports, nivolumab, surgery, cervix uteri, melanoma

## Abstract

Melanoma of the uterine cervix is an exceedingly rare malignancy that has high recurrence rates and distant metastases. In general, surgery is the preferred treatment for this tumor, and depending on stage additional consideration to radiotherapy and chemotherapy. Immunotherapy has emerged as a new treatment option in this context. The aim of this study was to report a case of melanoma of the uterine cervix that progressed rapidly to death while the patient was undergoing immunotherapy with nivolumab. A 39-year-old woman presented with an amelanotic ulcerated lesion of the uterine cervix in February of 2023. Histopathology study demonstrated melanoma of the uterine cervix. Treatment was initiated with surgery. Two months after cancer diagnosis, the tumor board decided to initiate adjuvant treatment with nivolumab. After four cycles of immunotherapy, progression of the disease occurred with death of the patient within six months of follow-up. The rare case presented illustrates the aggressive natural history of the tumor and possible use of immunotherapy in this context, despite current evidence that response to nivolumab is less effective in cervical melanoma than in skin melanoma.

## Introduction

Skin cancers are the most commonly diagnosed group of cancers worldwide, with more than 1.5 million new cases estimated in 2020. Malignant melanomas account for approximately 20% of these cancers, with approximately 325,000 cases and 57,000 deaths estimated globally in 2020 [1]. Although a common form of cancer, melanoma is exceedingly rare within the uterine cervix as the primary anatomic site and possibly due to late detection and advanced stage carries a very poor prognosis [1,2]. This tumor is thought to arise within melanocytes or melanocytic neural crest stem cells resident within the mucosal epithelium of the uterine cervix. Surgery, associated or not with radiotherapy and chemotherapy, is the most common approach to treatment. More recently, immunotherapy has also been used as an option, particularly in advanced disease [2].

The aim of this study was to report a case of melanoma of the uterine cervix that progressed rapidly to death while the patient was undergoing immunotherapy with nivolumab.

## Case presentation

On February 6, 2023, a 39-year-old female patient sought medical care after several days of abnormal genital bleeding and foul-smelling vaginal discharge. Personal medical and surgical history included only two cesarean sections and tubal ligation. Family medical history was obtained and revealed that her father had previously undergone treatment for colon cancer.

**Figure 1:**
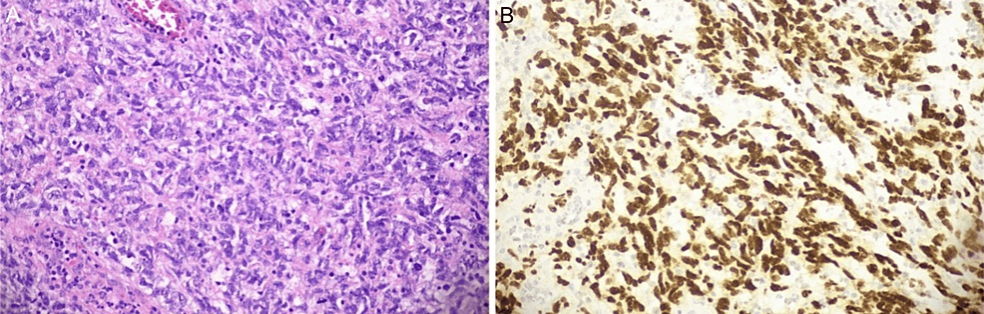
(A): Hematoxylin and eosin histopathology of lesion biopsy. (B): Immunohistochemistry of a lesion positive for SOX10. Magnification: x200.

On physical examination, an amelanotic ulcerated cervical lesion, measuring about 5 cm was found, mainly in the endocervix. The parametrium and pouch of Douglas were clear. Cervical vaginal cytology was normal and video colposcopy showed an ulcerated endocervical lesion. Biopsy of the lesion was performed and a poorly differentiated malignant tumor infiltrating the uterine cervix was diagnosed. Immunohistochemical study was inconclusive for tumor classification. A new biopsy was performed in the operating room for removal of a larger tumor sample and hematoxylin-eosin histopathology (Figure 1A). Immunohistochemistry (IHC) revealed epithelioid and atypical spindle cells with frequent mitotic figures and necrotic foci with strong, diffuse SOX10 immunohistochemical staining (Figure 1B). IHC was also positive for S100 and showed negativity for AE1/AE3, CD34, desmin, myogenin, p63, Melan-A, HMB-45, and a high molecular weight cytokeratin cocktail (34BE12). The fluorescence in situ hybridization (FISH) was negative for EWSR1 gene, ruling out the differential diagnosis of malignant gastrointestinal neuroectodermal tumor (GNET)/clear cell sarcoma. The final diagnosis was cervical melanoma.

**Figure 2:**
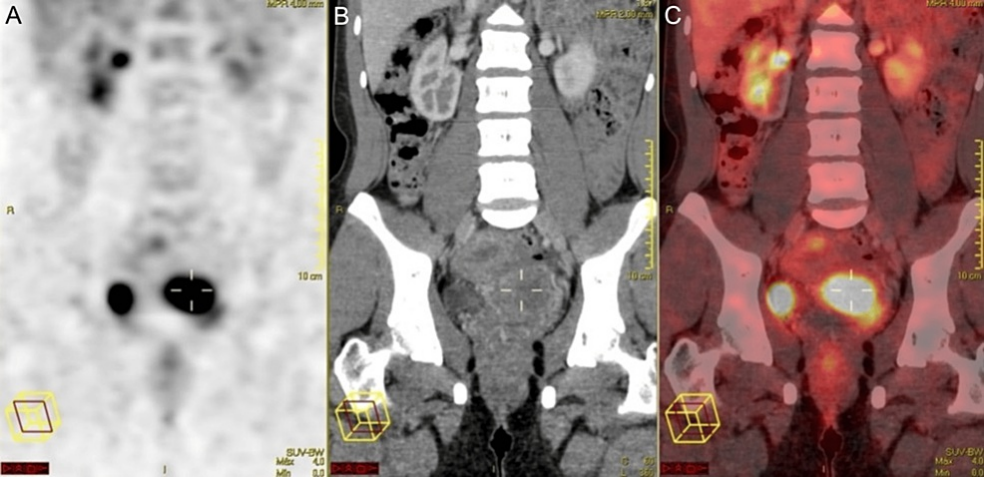
(A): Positron emission tomography scan (PET). (B): Computed tomography scan (CT). (C): Combined PET/CT scan.

Magnetic resonance imaging showed a cervical tumor, measuring 5x4 cm, with parametrial involvement and no suspicious lymph nodes in the vaginal vault. Chest and upper abdominal CT scans were normal, with a small nonspecific lesion in segment VI, measuring 0.7 cm, resulting in the diagnosis of melanoma. There was no evidence of metastases. Positron emission tomography scan (PET-CT) showed a cervical lesion, measuring 6 cm (maximum SUV: 14). Distant metastases were not revealed (Figure 2).

To rule out metastatic melanoma to the uterine cervix, dermatological and ophthalmological exams did not find any skin or mucosal lesions that were suspicious for melanoma.

With the diagnosis of melanoma confined to the uterine cervix, surgical treatment was indicated. The patient underwent radical hysterectomy with bilateral salpingo-ophorectomy, colpectomy and bilateral pelvic and para-aortic lymph node dissection, through a laparotomy. Intraoperatively, there was no evidence of metastatic disease in the pelvic and abdominal cavity, liver and diaphragmatic surface. Cytology of the peritoneal fluid was negative. Tumor stage was classified as FIGO IIB (pathologic staging).

In the postoperative period, the patient developed bowel subocclusion with good clinical resolution of the condition. Readmission to the hospital was due to urinary infection, treated successfully for seven days with ceftriaxone.

**Figure 3:**
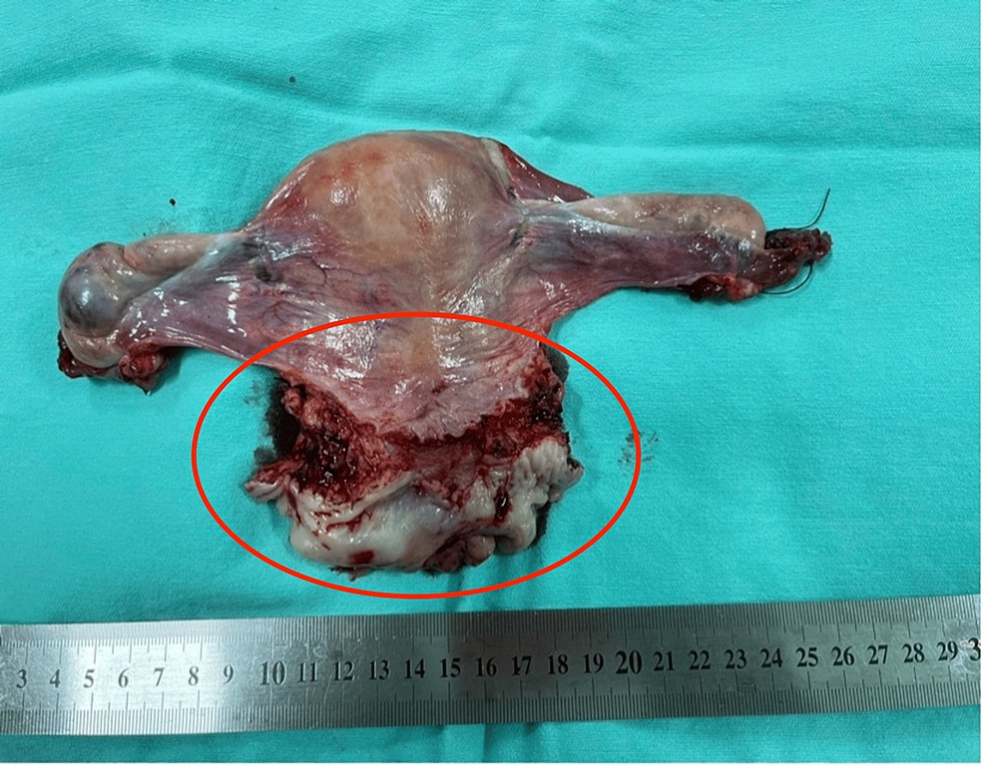
Surgical specimen of the patient. The circled region corresponds to that affected by tumor.

The final histopathology study of the surgical specimen (Figure 3) revealed an ulcerated epithelioid tumor of the uterine cervix, measuring 6.2 cm x 5.1 cm x 3.7 cm. Tumor depth of invasion was 28 mm, with no angiolymphatic or perineural invasion and free margins. The uterine body and ovaries revealed no lesions. Vaginal margins were cancer-free. Twelve lymph nodes were dissected, and all were clear of cancer cells.

Two months after diagnosis, a tumor board meeting determined that the patient would initiate nivolumab immunotherapy. After four cycles of immunotherapy, extensive pelvic tumor recurrence was observed. The patient progressed with poor general health and anemia, culminating in her death four months afterwards.

## Discussion

Melanoma of the uterine cervix is a very rare disease. In a review published in 2012, there were only 149 cases of cervical melanoma reported in the literature. The prognosis is usually poor, as shown in the present case [3].

Diagnosis of melanoma of the uterine cervix may be challenging to the pathologist, as described in this report. In the histopathology study, analysis of gene EWSR1 is useful to rule out sarcomas and GNET, as demonstrated in the present case. To confirm the diagnosis of primary cervical melanoma, skin, mucosal and ocular melanoma must be excluded. Specific clinical tests were used to rule out these other types of melanomas. In addition, a PET-CT scan did not demonstrate any other site with fluorodeoxyglucose (FDG) uptake. Metastasis from cervical melanoma is a very rare occurrence and the tumor is diagnosed by oncotic cytology and biopsy. In general, the primary tumor is already known or discovered by a complete physical exam, with the help of imaging tests [4].

Following surgical treatment, involving radical hysterectomy and lymph node dissection, the patient suffered complications including urinary tract infection and bowel sub-occlusion. Despite a short readmission to the hospital, she was doing well and was waiting for FISH test result.

Therefore, following case discussion, it was decided that immunotherapy with PD-1 inhibitor (nivolumab) 240 mg every two weeks would be administered after a two-month delay. However, after four cycles of immunotherapy, the patient had extensive pelvic recurrence, resulting in poor general health status, anemia and infection. She received supportive care and antibiotic therapy until death, which occurred six months after cancer diagnosis.

Melanoma of the uterine cervix has a high recurrence rate and metastatic dissemination. The five-year survival rate varies from 5% to 25%. The tumor usually manifests itself after the sixth decade of life. Abnormal vaginal bleeding is commonly the first symptom of the disease. In the literature, few cases have been reported. Nevertheless, an analysis was conducted by means of a systematic review, encompassing data until June 2022. This review included 96 case reports involving 137 patients diagnosed with metastatic cervical melanoma. Mean patient age was 56.5 years (range: 33 to 88 years) [2].

In a study by Kechagias et al., menopause status from 98 patients included 15 premenopausal and 83 postmenopausal women [2]. The most common symptom was vaginal bleeding, reported by 83% of the patients. For diagnosis, a biopsy (excisional or punch) was frequently used in 67% of the cases, followed by cytology in 18%. FIGO staging classification for cervical cancer was applied in 74 cases. Stage I was the most common (38%) classification, followed by Stages II (36%), III (19%) and IV (7%) [2]. In the present case, cervical cancer was FIGO Stage IIB, i.e., locally advanced tumor.

The main therapeutic approach was surgical intervention, which was performed in 90% of the cases. Hysterectomy (radical or total) was the most common procedure performed in 40% of these cases, along with salpingo-ophorectomy, with or without lymphadenectomy. Of the 105 patients with clinical outcome data, 58% died and 42% survived [2]. It is worth mentioning that the role of immunotherapy in cervical melanoma has still not been fully defined, due to the rarity of the disease. A search strategy for cases in PubMed was conducted, using the terms “nivolumab AND melanoma AND cervix AND case report”. Five cases of cervical melanoma treated with nivolumab immunotherapy were found. Of these, four cases evolved with rapid progression and death within a few months after diagnosis, as in the patient presented [5-11].

## Conclusions

The rare and challenging case of melanoma of the uterine cervix raises the possibility of using immunotherapy as a new therapeutic strategy for this type of cancer. However, the understanding of this treatment strategy is still an ongoing process. Furthermore, cervical melanoma has a poor prognosis, owing to high recurrence rates and distant metastases. This case report described rapid disease progression and patient death within six months of follow-up. It is worth highlighting that cervical melanoma has a less effective response to nivolumab than skin melanoma, according to data from the current literature.
